# Gadolinium and Bio-Metal Association: A Concentration Dependency Tested in a Renal Allograft and Investigated by Micro-Synchrotron XRF

**DOI:** 10.3390/jimaging8100254

**Published:** 2022-09-21

**Authors:** Wolf Osterode, Gerald Falkenberg, Heinz Regele

**Affiliations:** 1 Universitätsklinik für Innere Medizin II, Medical University of Vienna, A-1090 Vienna, Austria; 2 Photon Science, Deutsches Elektronen-Synchrotron (DESY), D-22603 Hamburg, Germany; 3 Klinisches Institut für Klinische Pathologie, Medical University of Vienna, A-1090 Vienna, Austria

**Keywords:** gadolinium, kidney, synchrotron X-ray fluorescence, zinc, iron, bromine

## Abstract

Aims: This study aimed to investigate gadolinium (Gd) and bio-metals in a renal allograft of a patient who was shortly after transplantation repeatedly exposed to a Gd-based contrast agent (GBCA), with the purpose of determining whether Gd can be proven and spatially and quantitatively imaged. Further elemental associations between Gd and bio-metals were also investigated. Materials and Methods: Archival paraffin-embedded kidney tissue (eight weeks after transplantation) was investigated by microscopic synchrotron X-ray fluorescence (µSRXRF) at the DORIS III storage ring, beamline L, at HASYLAB/DESY (Hamburg, Germany). For the quantification of elements, X-ray spectra were peak-fitted, and the net peak intensities were normalized to the intensity of the incoming monochromatic beam intensity. Concentrations were calculated by fundamental parameter-based program quant and external standardization. Results: Analysis of about 15,000 µSRXRF spectra (comprising allograft tissue of four cm^2^) Gd distribution could be quantitatively demonstrated in a near histological resolution. Mean Gd resulted in 24 ± 55 ppm with a maximum of 2363 ppm. The standard deviation of ±55 ppm characterized the huge differences in Gd and not in detection accuracy. Gd was heterogeneously but not randomly distributed and was mostly found in areas with interstitial fibrosis and tubular atrophy. Concentrations of all other investigated elements in the allograft resembled those found in normal kidney tissue. No correlations between Gd and bio-metals such as calcium, strontium or zinc below ~40 ppm Gd existed. In areas with extremely high Gd, Gd was associated with iron and zinc. Conclusions: We could show that no dose-dependent association between Gd and bio-metals exists—least in renal tissue—at Gd concentrations below ~40 ppm Gd. This was proven compared with a GBCA-exposed end-stage renal failure in which the mean Gd was ten-fold higher. Our results could shed additional light on Gd metabolism.

## 1. Introduction

Gadolinium-based contrast agents (GBCAs) are widely used to improve magnetic resonance imaging (MRI) results. In healthy volunteers, according to pharmacokinetic studies, GBCA’s half-life is about 1.3 h [1]. However, independently of renal function, linear or macrocyclic GBCAs are not readily and completely excreted by the hepatobiliary or renal system, and they are suspected to be responsible for inducing toxic effects, which result in the worst case in nephrogenic systemic fibrosis (NSF) [2,3,4,5,6]. On the other hand, renal function can affect the rate of Gd deposition [7,8,9,10]. Physiologically seen delayed GBCA clearance seems to result in greater dissociation of Gd3^+^ ions from the ligand, leading to subsequent accumulation in different tissues. Free Gd3^+^ ions are suspected to be co-initiators for fibrosis [11], and competition with biological and endogenous ions can thus lead to dechelation of gadolinium from a complex by inducing trans-metalation [12,13,14]. Metals that seem to be involved in trans-metalation are calcium (Ca), zinc (Zn), copper (Cu), and iron (Fe) [15,16]. In a recent paper, we could document strong associations between Gd and such elements in the renal tissue of a patient with end-stage renal failure [17]. That patient was exposed to GBCA three times within 16 months. We therefore determined whether such correlations exist in normal renal tissue after repeated Gd exposure as well.

For this reason, we retrospectively investigated archival renal biopsies from a patient with an allograft that was repeatedly exposed to GBCA within only 45 days. Since the allograft was not exposed to GBCA before we determined whether after such a short time of exposure Gd can be documented and whether competition with biological and endogenous ions exist, presuming that ion tissue concentrations and time of exposure may play an essential role. 

## 2. Materials and Methods

### 2.1. Control Kidney Tissue (C-KT)

Archival paraffin-embedded blocks of kidney tissue from 4 patients (age: 51 ± 5 years) who died from cardiovascular failure and with no known GBCA application were investigated. The size of paraffin blocks was about 2 × 2 × 0.5 cm^3^. Slices from these blocks with 10 µm thickness were investigated by micro-Synchrotron X-ray Fluorescence (Table 1).

### 2.2. Patient and Tissue Specimen (P-KT)

Archival paraffin-embedded tissue (Needle Biopsy) from a male patient 41 years of age was retrospectively re-investigated. He received a renal allograft after initial renal failure due to severe glomerulonephritis. About 8 weeks before the investigated biopsy, the patient underwent a kidney allograft. Needle biopsy four weeks later revealed slight intimal fibrosis and slight tubular atrophy, but inconspicuous glomeruli and no signs of rejection. 

**Figure 1:**
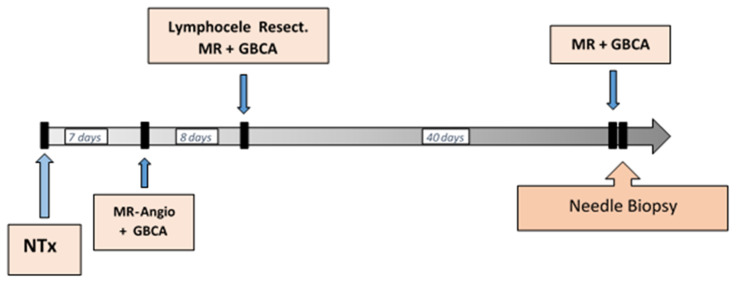
Timeline of clinical investigations with GBCA applications until needle biopsy.

Two months after transplantation, he had a GBCA-MRI (Gadodiamide) and subsequent resection of a lymphocele left thigh, showing histologically lamellar layered connective tissue with purulent inflammation, partial fibrosis, and bleeding. Since, meanwhile, the creatinine increased from 2.3 to 3.8 mg/dL and the GBCA-MRI was not conclusive, a kidney biopsy was performed, diagnosing an interstitial transplant rejection Type 1A BANFF 97 with slight TX glomerulitis, slight interstitial fibrosis, and subscapular tubular atrophy. A timeline of GBCA applications until the investigated needle biopsy is given in Figure 1.

### 2.3. Tissue Preparation for µSRXRF Investigation

Slices with 10 μm thickness were cut from the paraffin-embedded kidney specimens by a metal-free microtome knife and fixed (only by adhesion) on a trace-element-free Ultralene Kapton foil^®^ (SPEX SamplePrep, Metuchen, NJ, USA) that was 4 μm in thickness for µSRXRF investigation [17,18,19]. These were about 30 µm apart from those for histological inspection.

### 2.4. Microscopic Synchrotron Radiation X-ray Fluorescence Analysis

We have extensively described the technical setup used for the performed measurements in recent papers [19]. In short, microscopic synchrotron radiation X-ray fluorescence analysis (μ-SRXRF) measurements were performed at beamline L at the DORIS III storage ring at HASYLAB/DESY in Hamburg. Synchrotron radiation from a bending magnet was monochromatized by a NiC multilayer monochomator at 17 keV. A polycapillary optic was used to focus the beam to about 15 micrometres in diameter (FWHM) at a flux of 10^11^ photons/s. Kidney slices were mounted on an XYZ-sample stage oriented at 45 degrees towards the beam and were scanned through the X-ray micro-beam in on-the-fly mode at room temperature and in an air environment; therefore, light elements, such as Na and phosphorus (P), could not reliably be detected, and they are not given in the tables. In the present study, a constant sample dwell time of 2 s per point was used. The increment in the horizontal and vertical direction was 15 μm. Fluorescence photons and scattered radiation were detected perpendicular to the X-ray beam by an energy-dispersive silicon drift detector (Vortex 90, Hitachi High-Tech in America, Schaumburg, IL, USA). The detector was equipped with a Ta collimator of a 3 mm diameter opening. The collimator opening had a distance of 10 mm to the sample and 15 mm to the detector sensor.

### 2.5. Quantification of Elements

For the (semi-)quantification of element concentrations, X-ray spectra were peak-fitted, using the AXIL program package, in order to extract the net intensities of X-ray fluorescence lines [19]. The net peak intensities were normalized to the intensity of the incoming monochromatic beam intensity and sample dwell time. A germanium standard foil of homogeneous Ge area density of 2.6 × 10^−7^ g/cm^2^ was employed for external standardization [20]. Element concentrations were calculated from the normalized net peak intensities using the fundamental-parameter-method-based program package “quant” (mean sample mass density 1.1 g/cm^3^). Sample thickness, measurement geometry, and beam energy were unchanged during the measurement series. For all measurements, the same set of element-specific conversion factors from line intensities to concentrations were applied. For elements under investigation, absorption effects could be neglected. Table 2 shows the results of the determined element concentrations in the allograft.

### 2.6. Statistical Analysis

Mean values for investigated elements within scanned areas were calculated. Results are reported as mean ± SD. Tissue-empty areas within scanned areas were not considered for mean value calculations. SD values in this data management do not represent the precision of evaluated Gd concentrations or other elements but essentially the variance of the elemental distribution.

## 3. Results

**Figure 2:**
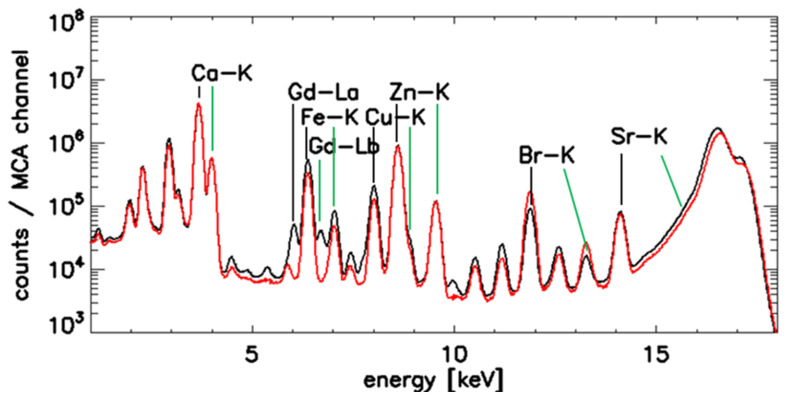
Accumulated SRXRF spectra of scans of control kidney (red line) and kidney tissue from a patient with GBCA application (black line). Alpha lines are annotated by vertical black lines and beta peaks by green lines. Note the Gd–Lα and Lβ peaks, which are not detectable in the spectrum of the control sample. Peaks of the Gadolinium L series (Gd-Lα and Gd-Lβ) are clearly resolved in the GBCA-exposed kidney, but are not detectable in the kidney not exposed to GBCA.

X-ray spectra summed over all scan pixel of the GBCA-exposed kidney biopsy (solid line) and of a control kidney not exposed to GBCA (dotted line) are shown in Figure 2. 

**Figure 3:**
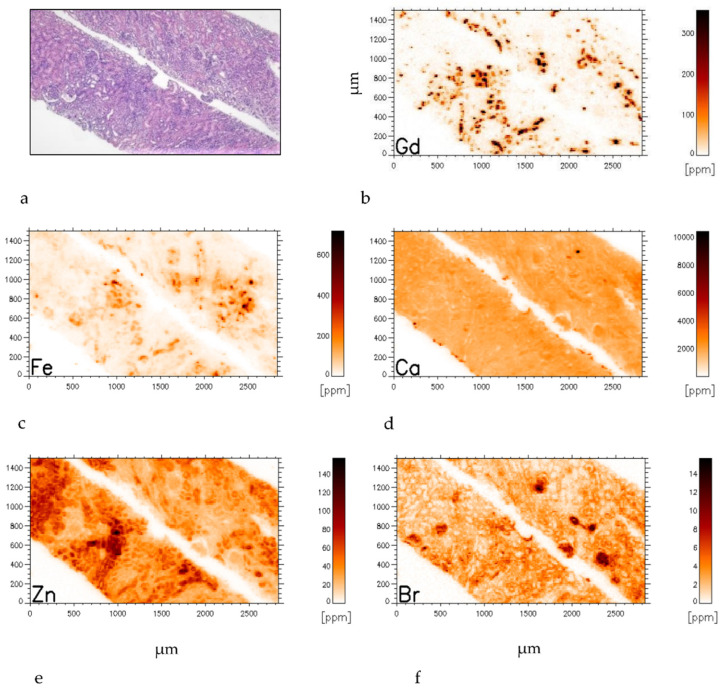
Needle biopsy of the GBCA exposed renal allograft with quantified bio-metal distribution within tissue (**a**–**f**). (**a**) Histological section of the needle biopsy (HE: hematoxylin-eosin staining). (**b**) Quantified gadolinium mapping of the same area as the parallel section shown in Figure 3a (about 30 µm apart). (**c**) Iron distribution, (**d**) calcium distribution, (**e**) zinc distribution, and (**f**) bromine distribution within the investigated area. The colour code indicates the element concentration in ppm in each element map.

Two-dimensional distributions of the investigated bio-metals are given in Figure 3 for comparison with the histological section of the GBCA-exposed renal biopsy. The histological section and the corresponding section for SRXRF investigation are only about 30 µm apart. 

**Figure 4:**
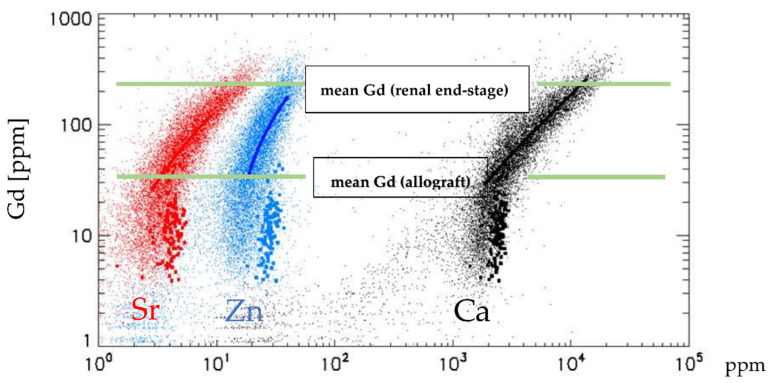
Comparison of element concentrations versus Gd in an end-stage renal failure and Gd in the renal allograft in a double logarithmic scale. Both specimens were exposed to GBCA three times. Small dots in the scatterplot characterize Gd vs. strontium (Sr, red), zinc (Zn, blue), and calcium (Ca, black) in end-stage renal failure (data are adapted with permission from our recent publication [17]), while the thick dots in the scattergram represent their distribution vs. Gd in the allograft. Red, blue, and black lines show correlations between Gd and elements, which are not given for Gd <~40 ppm. Green lines show the mean Gd concentrations in the end-stage renal failure and the allograft with nearly normal renal tissue, respectively. The mean Gd concentration in a renal allograft is about ten times lower than in end-stage renal failure. Both investigations were performed at the same µSRXRF facility.

The Gd distribution can be described as a constant background level of about 8 ppm Gd with some 10 micrometre patches of highly elevated concentration in a netlike arrangement. Overall, 14,933 single spectra were analysed for plotting the two-dimensional distributions of Gd and co-elements (Figure 4). The mean concentration of Gd in our specimen is 24 ± 55 ppm with a maximum of 2363 ppm (Table 2). The other investigated element levels (Ca, Zn, Fe, Sr, Cu) nearly resembled those found in the control kidney tissue not exposed to GBCA (Table 1). Empty tissue regions were excluded from the calculations.

In contrast to our findings in a prior investigation of an end-stage kidney [17], Ca, Sr, and Cu were homogeneously distributed. Only Fe and Zn seem to be structurally emphasized and associated with fibrotic areas (Figure 3c,e) Our data seem to confirm that in totally preserved tubule areas, Gd can readily be excreted and will not be stored, while in fibrotic tissue, Gd deposition is enhanced (Figure 3b,c). We have previously shown that bromine (Br) can characterize connective tissue [17,18,21,22]. Therefore, its distribution is also displayed in order to highlight fibrotic areas (Figure 3f). In preserved tubules, Br is low (Figure 3f left upper corner) however, Br characterizes glomeruli (Figure 3f) which matches with those in the histological HE staining (Figure 3a).

**Figure 5:**
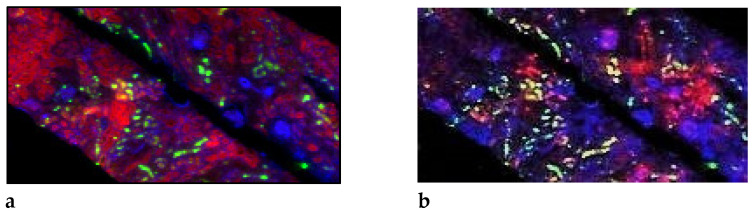
Superimposed gadolinium and bio-element distribution by a RGB image representation. (**a**): Superposition of Zn (red), Gd (green), and Br (blue) distributions demonstrates only a slight association between Zn and Gd (red + green = yellow), but not in the totally preserved tubule in the left upper corner. (**b**) Superposition of Fe (red), Gd (green), and again Br (blue). Blue spots (Br) characterize glomeruli and correspond to those in Figure 3a. x-axis: 0–2800 µm, y-axis 0–1500 µm.

The mean Gd concentration in the tissue of this recently transplanted kidney was about ten times lower than in our previous Gd investigation in a fibrotic end-stage kidney. Furthermore, correlations between Gd/Ca, Gd/Sr, and Gd/Zn could not be established. Nevertheless, since Gd, like Fe and Zn, was heterogeneously distributed, we visualized possible co-depositions of Gd with other elements. This is shown by overlaying Zn, Gd, and Br (coloured red, green, and blue) concentrations. In Figure 5a, slight local association between Zn and Gd (red + green = yellow) depositions exist, while superimposed Zn, Gd, and Fe are visualized in Figure 5b. In areas with totally preserved tubule (left upper corner Figure 3a), no such associations exist. The same seems to be valid for Gd and Fe (Figure 5b).

A side note is that we had the opportunity to investigate the tissue of the lymphocele resection of the same patient, and interestingly, small amounts of Gd were detectable in granulation tissue of the lymphocele even eight days after GBCA application (Figure 1).

## 4. Discussion

We were able to show that after a cumulative GBCA load within 6 weeks (Figure 1), mean Gd concentration in the renal allograft was 24 ± 55 ppm. Compared to our previous findings in a kidney with end-stage renal failure that was also cumulatively exposed to Gd—although within 16 months—Gd is about 10 times lower [17]. The allograft had not been GBCA-exposed before. We assume that in a recently transplanted kidney with essentially preserved histological architecture, GBCA excretion is widely preserved, and the deposition of intra- or extracellular Gd-complexes is therefore lower with respect to end-stage renal failure. Gd was heterogeneously distributed (Figure 3b). In preserved tubular areas, Gd was not detectable or was below our detection limit of 5–10 ppm, while in areas with slight interstitial fibrosis and tubular atrophy, Gd could be documented. Nonetheless, the mean Gd of 24 ppm seems to be high. By exploring the prehistory of the allograft, we figured out that the patient was exposed to a linear GBCA. Linear GBCAs result in more gadolinium retention and are less stable than macrocyclic GBCAs [23,24,25,26]. This may have contributed to such Gd concentrations. However, even for macrolytic GBCAs, great differences exist in terms of their propensities to accumulate in tissues [27]. Although experiments were performed mostly in mice or rats, we may assume similarities in humans.

A central question of this investigation was whether a concentration dependency or dose dependency exists between Gd and bio-metals. Endogenous cations are involved in the displacement of Gd from a GBCA or ligand, inducing trans-metalations [13]. Using different techniques, Gd was often associated with calcium that has nearly the same ionic radius as Gd, but zinc and/or copper serve(s) as a stronger participant than calcium in a trans-metalation reaction. The deposition of various Gd complexes may be tissue-dependent and, dependent on the range of available endogenous ions such as zinc and calcium [28,29]. In a recent paper, we showed—maybe for the first time—a strong dose dependency between Gd and Ca, Zn, and Sr. However, these dependencies existed only at Gd >~40 ppm [17]. In our allograft with a mean Gd of 24 ± 55 ppm, no such associations existed, which confirms our previous finding (Figure 5) that high tissue Gd promotes associations with cations. To what extent a time factor or different tissues play a role in building such Gd complexes remains open.

In slightly fibrotic areas, zinc and iron were unevenly dispersed (Figure 3c and Figure 4e). In particular, in fibrotic tissue, depositions of insoluble Gd complexes have been repeatedly reported [30]. Such Gd complexes are mainly composed of Gd, phosphorus (P), calcium (Ca), and light elements. Therefore, George et al., 2010 [31], used SRXRF and extended absorption fine structure (EXAFS) spectroscopy and SEM/EDS analysis in parallel to demonstrate such Gd complexes in the skin of a patients with documented NSF. With our SRXRF device, phosphorus could not be reliably detected on a single scan-point level due to low statistics for the P peak adjacent to the Ca escape peak.

Concerning the extreme Gd-SD—which of course does not represent the accuracy of our µSRXRF measurements (Table 2), but indicates a huge Gd variance within our tissue sample—we calculated a Gd-maximum of 2363 ppm, a concentration about a hundred times higher than the mean value. Locally high Gd values were found not only in our recent paper, but also by Wang et al., 2019 [27], in brain tissue after repeated GBCA application in mice. Interestingly, they found concentrations up to 5894 µg Gd/g tissue in areas with high inflammatory activity. This may correspond to perivascular areas of inflammation in our images. By means of nano-SRXRF, these hotspots were located in areas with a diameter of ~160 nm. Our Gd-max concentrations are valid for areas of 10 × 10 µm^2^. Therefore, our concentrations are averaged in a larger area and thus under-calculated compared to a compartment of 160 nm in diameter. We assume that in different tissues, including kidney, the pathophysiological and bio-chemical mechanisms are comparable. However, a conclusive theory that could explain such Gd hotspots is currently not available. Moreover, these findings seem to support the observation that only Gd in high concentrations can form Gd complexes with endogenous ions.

Since the distribution of Gd does not totally match with fibrotic tissue, we suggest that superimposing the spatial distribution of elements gives an impression of associations between elements. Superposing, e.g., Zn, Gd, and Bromine (Br) demonstrates only a slight association between Zn and Gd (red + green = yellow), while in totally preserved tubuli (upper-left corner), Zn dominates (Figure 5a). Figure 5b shows the superposition of Fe (red), Gd (green), and again Br (blue). We find that Gd is more closely associated with Fe than with Zn.

As documented earlier, the distribution of Br characterizes connective and interstitial tissue and thus, in part, fibrotic tissue as well [17,18,21,22]. While Br was lower in the preserved renal architecture, it was higher in regions with fibrosis and tubular atrophy. Moreover, glomeruli are clearly defined by Br (Figure 3f), which seems to be in line with the histological description of slight glomerulonephritis. If Br is tracing fibrosis—fibrosis is not part of glomerulonephritis until the sclerotic stage—this finding seems to be interesting for further studies.

The principal limitation of this study is that only one GBCA-exposed (allograft) kidney tissue could be investigated and that only formalin-fixed paraffin-embedded tissue and not shock-frozen sections were available. Shock-frozen-hydrated tissue probes are now the generally accepted and recommended method of sample preparation [32,33,34]. To estimate the effect of tissue preparation, paired liver tissues (shock-frozen/paraffin) were prepared. Chlorine and potassium were increased in shock-frozen samples. Cu in paraffin sections was increased by 20–30%, while Fe and Zn was reduced by 10–15% (personal communication W.O. and G.F.). Paraffin-embedded tissue of the control kidney and GBCA kidney was processed using the same method and on the same tissue type so that the partial washing out of their elements was similar. Lastly, the main interest in this study was Cu, Zn, and Fe, metals that are normally bound to proteins in tissue. Thus, we may assume that, on the tissue level, their distribution is maintained. Since µSRXRF sensitively detects elements but does not identify molecule complexes in which Gd is incorporated, only allocative assumptions were made.

In summary, we demonstrated that in a recently transplanted renal allograft, remarkable Gd concentrations were provable at a near-histological resolution. Gd was heterogeneously distributed. While Gd was not verifiable in preserved renal tissue, in fibrotic areas and areas with tubule atrophy, Gd was detectable. The quantitative deposition of Gd in GBCA-affected tissue covered an extraordinary range of Gd > 2000 ppm. However, in contrast to our previous investigation, correlations between Gd and Ca, Zn, Cu, or Fe could not be established, assuming that a mean Gd of 24 ppm was too low to exhibit significant correlations between endogenous ions in general. The fact that sonly Gd tissue concentrations above ~40–50 ppm can lead to a dose-dependent association with tissue bio-metals is an interesting new aspect that could shed additional light on Gd metabolism.

## Figures and Tables

**Table 1:** Element concentrations in control kidney tissue.

Element [ppm]	Gd	Ca	Zn	Fe	Sr	Cu
**mean**	<LLD	2001	41	35	5	8
**SD**	<LLD	978	16	21	3	4
**Max**	<LLD	7266	123	237	11	35

Mean values of elements in ppm in control kidney tissue. SD: standard deviation, maximum values (max). <LLD: lower than the lowest limit of detection.

**Table 2:** Element concentrations in GBCA exposed needle biopsy tissue.

Element (ppm)	Gd	Ca	Zn	Fe	Sr	Cu
**mean**	24	1868	31	39	2	9
**SD**	55	726	20	41	1	22
**Max**	2363	10,449	157	720	38	964

Mean values of elements in ppm in kidney tissue exposed to GBCA. SD: standard deviation and maximum (max) values.

## Data Availability

Not applicable.

## Abbreviations

GBCA	Gadolinium-based contrast agents
Gd	Gadolinium
SRXRF	Synchrotron X-ray fluorescence
NSF	Nephrogenic systemic fibrosis
